# Revealing the Tick Microbiome: Insights into Midgut and Salivary Gland Microbiota of Female *Ixodes ricinus* Ticks

**DOI:** 10.3390/ijms24021100

**Published:** 2023-01-06

**Authors:** Anna Wiesinger, Jasmin Wenderlein, Sebastian Ulrich, Stephanie Hiereth, Lidia Chitimia-Dobler, Reinhard K. Straubinger

**Affiliations:** 1 Chair of Bacteriology and Mycology, Institute for Infectious Diseases and Zoonosis, Department of Veterinary Sciences, Faculty of Veterinary Medicine, LMU Munich, 80539 Munich, Germany; 2 Bundeswehr Institute of Microbiology (InstMikroBioBw), Neuherbergstraße 11, 80937 Munich, Germany

**Keywords:** *Ixodes ricinus*, microbiome, tick-borne disease, salivary glands, midgut, endosymbiont, *Candidatus* Midichloria mitochondrii

## Abstract

The ectoparasite *Ixodes ricinus* is an important vector for many tick-borne diseases (TBD) in the northern hemisphere, such as Lyme borreliosis, rickettsiosis, human granulocytic anaplasmosis, or tick-borne encephalitis virus. As climate change will lead to rising temperatures in the next years, we expect an increase in tick activity, tick population, and thus in the spread of TBD. Consequently, it has never been more critical to understand relationships within the microbial communities in ticks that might contribute to the tick’s fitness and the occurrence of TBD. Therefore, we analyzed the microbiota in different tick tissues such as midgut, salivary glands, and residual tick material, as well as the microbiota in complete *Ixodes ricinus* ticks using 16S rRNA gene amplicon sequencing. By using a newly developed DNA extraction protocol for tick tissue samples and a self-designed mock community, we were able to detect endosymbionts and pathogens that have been described in the literature previously. Further, this study displayed the usefulness of including a mock community during bioinformatic analysis to identify essential bacteria within the tick.

## 1. Introduction

*Ixodes ricinus* is the most common tick species in Europe [1] with a biphasic, seasonal activity pattern [2]. In spring, *I. ricinus* begins questing for hosts at temperatures above 7 °C [3,4] and stops in autumn when temperatures lower and days become shorter [5]. Then, the ticks go into diapause until temperatures rise again [6,7], surviving sub-zero temperatures as low as −10 °C [8]. The Fifth Assessment Report of the International Panel on Climate Change (IPCC) assumes a temperature increase of 0.9 to 2.3 °C by 2100, if climate policy is ambitious [9]. This assumption favors an increased reproduction of ticks over the next years, the distribution of hosts and attached ticks into regions and heights that were inhabitable before, and an associated increase in tick-borne disease (TBD) [10]. A connection between the increase of TBD occurrence and climate change has already been described in 2001 for Sweden [11]. In this work, the incidence of TBD was linked to five other variables: two mild winters in succession, temperatures favoring spring development, a long autumn in the previous year, temperatures favorable for ticks questing activity early in the year, and a deeper snow cover. Dautel et al. (2008) [7] first described the activity and the associated host questing of ticks in Germany in the winter of 2006 and 2007. Therefore, in mild winters, forests and park areas are possible locations for tick attachment and thus infections with TBD [7].

TBD include human- and animal-related pathogens such as *Borrelia* spp., *Anaplasma* spp., *Rickettsia* spp., *Candidatus* Neoehrlichia mikurensis, and tick-borne encephalitis virus. Lyme borreliosis (LB) is the most common bacterial tick-borne disease in the northern hemisphere [12]. This disease is caused by spirochetes from the *Borrelia burgdorferi* sensu lato (*Bb*sl) complex [13], and the estimated incidence in Bavaria (the location of tick collection in this study) is 40 cases per 100,000 inhabitants [14]. The causative agents of LB induce arthritis as well as neurological and cardiological symptoms in humans [13,15]. However, the diagnosis of this disease is not trivial. Early manifestations may be asymptomatic, nonspecific, or specific with the formation of *erythema migrans* [16]. Detection of the disease by serological methods is possible at the earliest three weeks after infection [17]. Furthermore, treatment of diagnosed LB is not always successful [18,19]. If elimination of borrelial organisms is not possible, the disease may convert into a chronic stage, characterized by alternating symptomatic and asymptomatic phases [20] with a significant reduction in the quality of life [21,22]. The disease’s progression and consequences, combined with the lack of vaccination for people, make it necessary to think about new ways to protect against infections with LB and further TBD.

To achieve a precise understanding of individual bacterial species, microorganisms need to be considered in their ecological context. Therefore, the bacterial community (BC) of the tick microbiome is in the focus of this research [23]. The microbiome is defined as the complete community of microorganisms and microbial metabolites, microbial structural elements, and the environmental conditions found in a specific location [24]. The part of the microbiome describing bacteria is defined as BC [24]. It describes single cells to multicellular aggregates and bacterial colonies. Moreover, the BC impacts tissues, organs, and eventually the entire host [25]. Knowledge on common BCs in ticks’ microbiome is the key to later identify and describe bacterial species that are endosymbiotic to the tick or even bacterial species symbiotic or competitive to pathogens. Endosymbionts are described as essential bacteria for the development of the host that could influence the host’s capacity and fitness [26] and thus the transmission of pathogens [27]. Symbiosis is defined as any type of a close and long-term biological interaction between two different biological organisms. Symbionts must be of a different species, and it does not matter whether this interaction is mutualistic, commensalistic, or parasitic [28]. Hence, identification and characterization of either endosymbionts, symbionts, or competitors within the ticks’ BCs might allow a precise manipulation of ticks intending to reduce the spread of TBD in tick populations and the transmission of pathogens to humans and animals.

However, knowledge on the *I. ricinus* tick’s microbiome regarding certain tissues is sparse, especially in Germany. There are several studies in Switzerland [29], the Netherlands [30], the Czech Republic [31], Spain [32], Ireland [33], and the US [34] describing the BCs of *Ixodes* spp. To the best of our knowledge, the microbiome of entire *I. ricinus* ticks in Germany has only been described by Hoffmann et al. (2021) [35]. Nevertheless, more comprehensive research on the tick’s microbiome concerning individual organs is lacking completely. Consequently, this study aims to define the BC in different tissue (i.e., in salivary glands, in the midgut, and in residual tick material) of *I. ricinus* ticks. Therefore, female ticks were collected, dissected under a stereomicroscope, and DNA was extracted for 16S rRNA gene amplicon sequencing.

## 2. Results

16S rRNA gene amplification produced a total of 7,847,782 sequences with an average of 37,018 sequences per sample (SD = 13,501) in the final analysis. The OTU (Operational Taxonomic Unit, i.e., molecular species) and zOTU (zero-radius Operational Taxonomic Unit, i.e., molecular strains) table contains a total of 459 OTUs and 882 zOTUs, respectively (Supplementary Tables S1 and S2).

**Figure 1:**
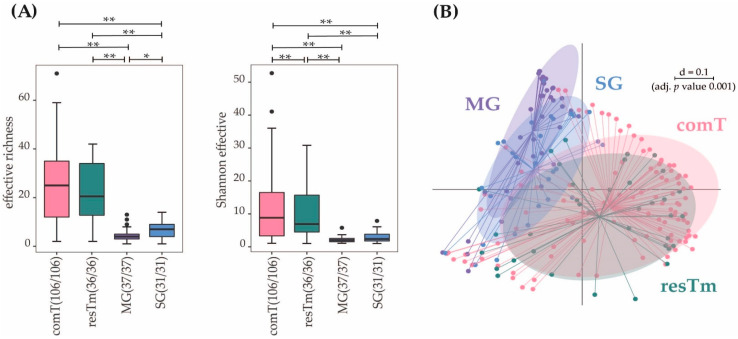
(**A**) α-diversity shown as effective richness and Shannon effective diversity between different tick tissue samples. Box-plots indicating median (thick bar), upper and lower quartile (within box), and standard deviation (whiskers). Outliers are depicted as dots. Brackets above the individual box-plots point to significance levels for pairwise comparison. (**B**) β-diversity of all sample types are displayed in a multidimensional-scaling (MDS) plot. The scale is an indicator for the differences in the phylogenetic makeup of microbiota between samples (β-diversity) based on general UniFrac distances (d = 0.1, 10% difference). comT, complete *Ixodes ricinus* tick; resTm, residual tick material; MG, midgut; SG, salivary gland. *p* value summary: * *p* ≤ 0.05, ** *p* ≤ 0.01.

Effective richness and Shannon effective diversity were calculated for α-diversity, and the results compared among complete *I. ricinus* ticks (comT) and different tick tissues’ samples (resTm, residual tick material; MG, midgut; SG, salivary glands; Table 1 and Figure 1A). Bacterial communities (BC) in complete ticks (comT) as well as in residual tick material samples from dissected ticks (resTm) were characterized by a higher count of bacterial species compared to specific organs such as midgut (MG) and salivary glands (SG). α-diversity in SG samples displayed a higher number of species when compared to MG.

The β-diversities of comT and resTm samples (i.e., comparing the microbial profiles between samples) overlapped almost completely, whereas samples from MG and SG differed most (Figure 1B). While SG samples still showed overlaps with comT and resTm samples, MG intersected only with SG samples.

**Figure 2:**
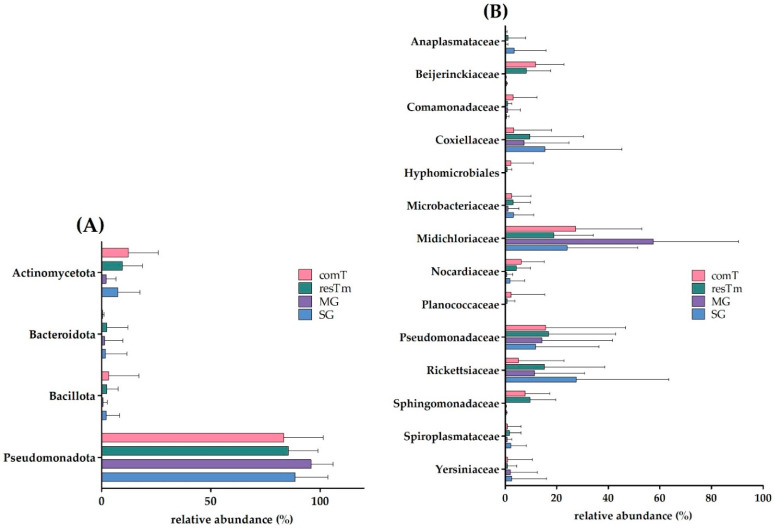
Bar-plots of (**A**) the four most abundant bacterial phyla and of (**B**) the fourteen most abundant bacterial families for all types of samples. The end of the bars displays the mean relative abundances (%), while the error bars indicate the standard deviation. comT, complete *Ixodes ricinus* tick; resTm, residual tick material; MG, midgut; SG, salivary gland.

At the phylum level, Pseudomonadota (previously Proteobacteria) were dominant in all types of samples. Strikingly, Actinomycetota (previously Actinobacteria) was the only phylum with a mean abundance above 7.5% present in SG, resTm, and comT samples. All other phyla were detected with an abundance of less than 7.5% (Figure 2A).

The dominant family in all samples, beside SG, was Midichloriaceae with an especially high abundance in MG samples (57.5%). Here, Pseudomonadaceae, Rickettsiaceae, and Coxiellaceae followed in the descending order. The second strongest abundance of Midichloriaceae was observed in comT samples (27.3%), followed by Pseudomonadaceae, Beijerinckiaceae, and Sphingomonadaceae. The next strongest abundance for Midichloriaceae was observed in SG samples (23.9%), however, Rickettsiaceae dominated this sample type. After those two families, Pseudomonadaceae, Coxiellaceae, and Anaplasmataceae followed. resTm samples included Midichloriaceae as the most abundant family (18.9%), followed by Pseudomonadaceae and Rickettsiaceae. Interestingly, the environmentally associated families Beijerinckiaceae and Sphingomonadaceae were only present in resTm and comT samples in an abundance above 0.5% (Figure 2B).

### 2.1. Complete Ixodes ricinus Tick Samples (comT)

**Figure 3:**
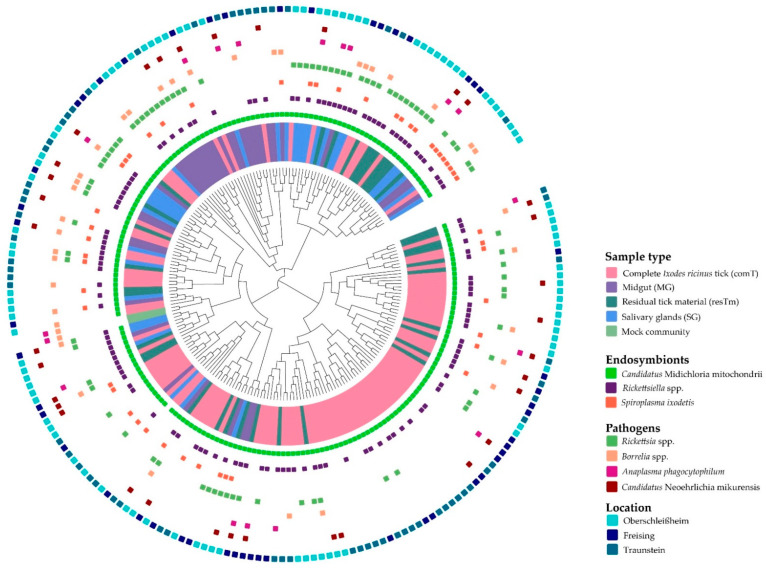
β-diversity is displayed as a circular dendrogram using a phylogenetic tree in the middle to demonstrate the genetic and taxonomic closeness and relationship, as well as the distribution of samples on phylogenetic branches. The innermost circle shows the different types of tissue highlighting their distribution. The next circles display the presence of probable endosymbionts and pathogenic bacteria in the tick microbiome. The outermost circle displays the location of the samples’ collection.

In comT samples, a mean of 32.8 effective species was calculated (Table 1, Figure 1A). The mean evenness of comT samples was 0.32 accounting for a stronger dominance of some species compared to the totality of species in this BC. The microbial profiles of comT samples displayed a strong heterogenicity (Figure 1B). In the dendrogram, comT samples mainly clustered on one branch, whereas many outliers appeared on the other two branches (Figure 3). As described above, comT samples were dominated by the phylum Pseudomonadota (83.2%), while at family level Midichloriaceae, Pseudomonadaceae, Beijerinckiaceae, Sphingomonadaceae, Nocardiaceae, and Rickettsiaceae were most abundant (abundance above 5.0%, Figure 2). At genus level, comT samples were dominated by *Candidatus* Midichloria, followed by *Pseudomonas*, *Methylobacterium*, *Sphingomonas*, and *Rickettsia*. At molecular strain level, the complete genus *Candidatus* Midichloria was represented by zOTU1 (*Candidatus* Midichloria mitochondrii, 100% similarity). The second most abundant molecular species was zOTU2 (*Rickettsia helvetica,* 100% similarity), representing the strongest fraction of the *Rickettsia* genus (4.9% of 5.1%). The genus *Pseudomonas* (*P*.) seemed to be composed of various molecular strains in abundances below 3.0%. The four most abundant molecular stains of this genus were zOTU4 (*P. extremaustralis*, 100% similarity), zOTU7 (*P. migulae*, 99.8% similarity), zOTU14 (*P. orientalis*, 99.8% similarity), and zOTU12 (*P. edaphica*, 99.8% similarity) in descending order (Supplementary Table S3). Interestingly, comT samples included a molecular strain (zOTU3, *Rickettsiella popilliae,* 98.4% similarity) of the genus *Rickettsiella*, which is thought to be an endosymbiont of *I. ricinus* ticks [36]. In total, eleven zOTUs displayed an abundance above 1.0% in comT samples of which all molecular strains not described above were environmentally associated according to literature.

### 2.2. Residual Tick Material Samples (resTm)

A mean of 28.3 effective species was calculated for resTm samples (Table 1). The mean evenness of resTm samples (0.32) equaled the mean evenness of comT samples, demonstrating again that only some species dominate this type of tissue. resTm samples showed a strong heterogenicity in their β-diversity (Figure 1B), which is most obvious on the dendrogram where resTm samples seemingly clustered randomly on all branches (Figure 3). In resTm samples, the dominant phylum was Pseudomonadota (85.4%). At the family level, Midichloriaceae was most abundant, followed by Pseudomonadaceae, Rickettsiaceae, Sphingomonadaceae, Coxiellaceae, and Beijernickiaceae in descending order (Figure 2). At genus level, resTm samples were again dominated by *Candidatus* Midichloria, followed by *Pseudomonas*, *Rickettsia*, *Rickettsiella*, *Sphingomonas*, and *Methylobacterium*.

At molecular strain level, the genus *Candidatus* Midichloria was represented only by zOTU1 (*Candidatus* Midichloria mitochondrii, 100% similarity). The next strongest molecular species was again zOTU2 (*Rickettsia helvetica*, 100% similarity), followed by two molecular strains of the genus *Rickettsiella* (zOTU3, *Rickettsiella popilliae*, 98.4% similarity and zOTU6, *Rickettsiella popilliae*, 98.7% similarity). Next, the genus *Pseudomonas* was again composed of various molecular strains with abundances below 2.0%. The four most abundant molecular strains of this genus were zOTU7 (*P. migulae*, 99.8% similarity), zOTU10 (*P. poea*, 100% similarity), zOTU11 (*P. synxantha*, 100% similarity), and zOTU4 (*P. extremaustralis*, 100% similarity) in descending order (Supplementary Table S3). Further, with an abundance above 1.0% zOTU20 (*Spiroplasma ixodetis*, 100% similarity), zOTU34 (*Rickettsiella viridis*, 96.6% similarity), and zOTU13 (*Candidatus* Neoehrlichia mikurensis, 98.4% similarity) occurred in resTm samples. In total, 18 zOTUs displayed an abundance above 1.0% in resTm samples. Molecular strains not described above are associated with the environment according to the literature (e.g., [37,38,39]).

**Figure 4:**
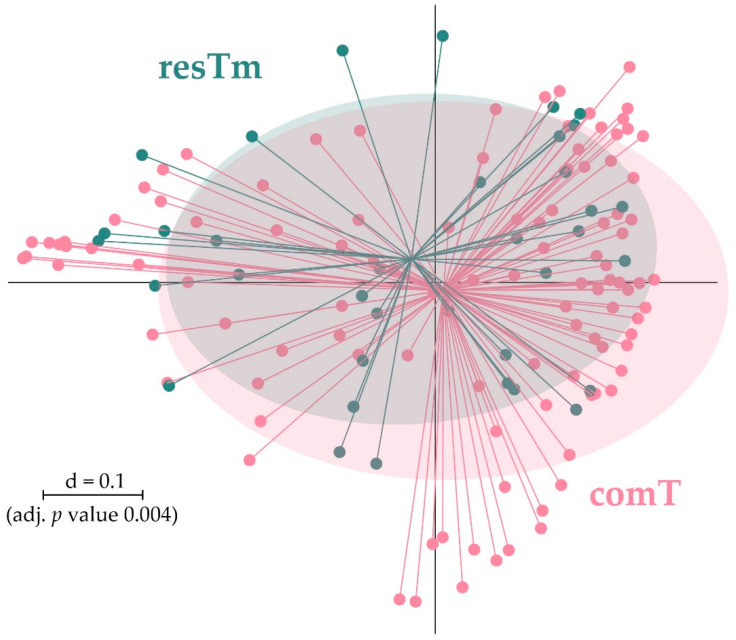
Pairwise β-diversity comparison of resTm and comT displayed as multidimensional-scaling (MDS) plot. The scale is an indicator for the differences in the phylogenetic makeup of microbiota between samples (β-diversity) based on general UniFrac distances (d = 0.1, 10% difference). resTm, residual tick material; comT, complete *Ixodes ricinus* tick.

comT and resTm samples displayed the highest bacterial diversity of all sample types. However, α-diversity parameters of resTm samples were slightly lower compared to comT samples (Figure 1A). Comparing comT and resTm microbial profiles in pairwise β-diversity, both groups apparently clustered close to each other; however, they differed significantly (Figure 4). As the displayed multi-dimensional scaling (MDS) plot is a two-dimensional representation of a three-dimensional plot, visual overlaps in this figure may not manifest in the third dimension. Nevertheless, the *p* value and the representation of samples in the dendrogram might allow more precise conclusions. Therefore, the difference between comT and resTm was more evident when considering the dendrogram (Figure 3).

**Figure 5:**
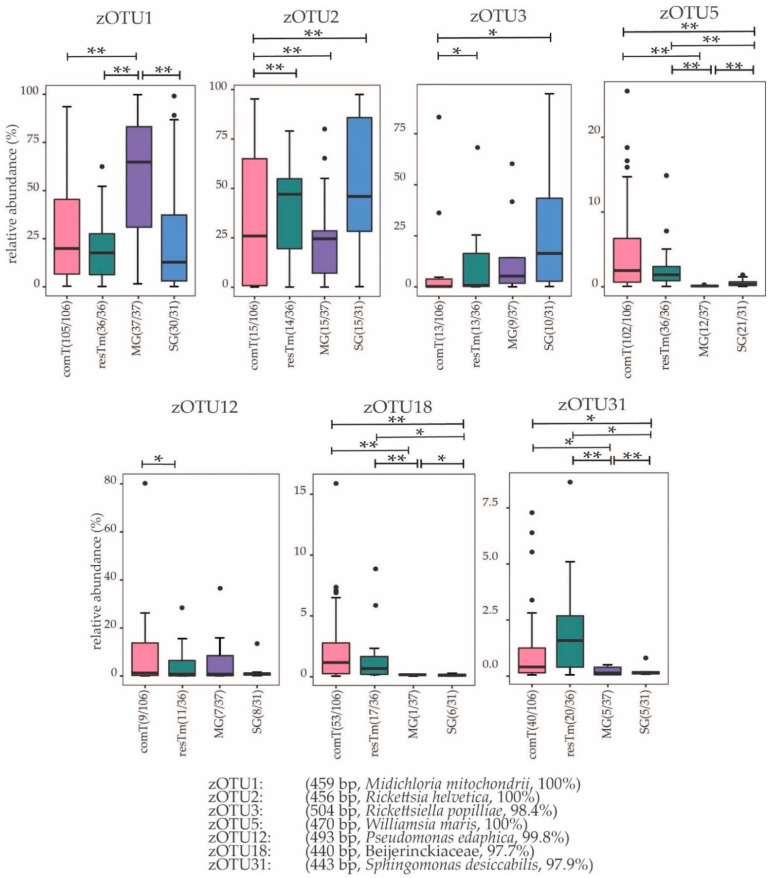
Box-blot representation of molecular strains relative abundance according to sample types. Box-plots visualize the median (thick bar), upper and lower quartile (within box), and standard deviation (whiskers). Outliers appear as dots. Brackets above the individual box-plots show significance for pairwise comparison. zOTUs were identified by EzBioCloud [40]; the sequence length, the closest relative taxon, and the sequence similarity score of zOTUs are shown in the order of appearance. zOTU: zero-radius operational taxonomic unit. comT, complete *Ixodes ricinus* tick; resTm, residual tick material; MG, midgut; SG, salivary gland. *p* value summary: * *p* ≤ 0.05, ** *p* ≤ 0.01.

When comT and resTm samples were compared, the genus *Rickettsia* and *Rickettsiella* were observed with a significantly higher abundance in resTm samples (*p* value ≤ 0.001 and *p* ≤ 0.05, respectively). This observation was also significant at the molecular strain level with zOTU2 (*Rickettsia helvetica*, 100% similarity) and zOTU3 (*Rickettsiella popilliae*, 98.4% similarity). Further, the order Hyphomicrobiales (*p* ≤ 0.05) with its genus *Bradyrhizobium* (*p* ≤ 0.001) were significantly higher and more frequent in comT than in resTm samples (Supplementary Table S4). zOTU12 (*P. edaphica*, 99.8% similarity) was significantly different in abundance between comT and resTm samples (Figure 5). In general, significant differences between comT and resTm need to be considered carefully as resTm represent pooled samples of three ticks and thus some bacterial species might appear overrepresented.

### 2.3. Midgut Samples (MG)

In MG samples, a mean of 4.9 effective species was obtained. MG samples displayed the lowest α-diversity of all sample types (Figure 1A). Further, its mean evenness was clearly lower than the evenness of comT and resTm samples. With a mean evenness of 0.2 even fewer species seemed to dominate this sample type. At molecular strain level, this was confirmed by an abundance of 57.5% of zOTU1 (*Candidatus* Midichloria mitochondrii, 100% similarity). The microbial profiles of MG samples displayed a strong heterogenicity with two clusters (Figure 1B); nevertheless, on the dendrogram most samples clustered on one branch (Figure 3). At phylum level, Pseudomonadota (95.8%) dominated the MG samples. As mentioned above, over the half of the BC from MG samples consisted of the family Midichloriaceae, followed by Pseudomonadaceae, Rickettsiaceae, and Coxiellaceae. These families were represented by only one genus, respectively (i.e., *Candidatus* Midichloria, *Pseudomonas*, *Rickettsia*, and *Rickettsiella*). As already described, zOTU1 (*Candidatus* Midichloria mitochondrii, 100% similarity) dominated the BC of MG samples. Further, the genus *Rickettsia* was again dominated by zOTU2 (*Rickettsia helvetica,* 100% similarity), likewise the genus *Rickettsiella* by zOTU3 (*Rickettsiella popilliae*, 98.4% similarity) and zOTU6 (*Rickettsiella popilliae*, 98.7% similarity) with a lower abundance. The genus *Pseudomonas* consisted of different molecular species (zOTU11, *P. synxantha*, 100% similarity; zOTU4, *P. extremaustralis*, 100% similarity; zOTU12, *P. edaphica*, 99.8% similarity; zOTU16, *P. lurida*, 100% similarity; Supplementary Table S3). In MG samples, zOTU20 (*Spiroplasma ixodetis*, 100% similarity) was observed as well. In total, eleven zOTUs displayed an abundance above 1.0% in MG samples of which all molecular strains not described above were environmentally associated.

**Figure 6:**
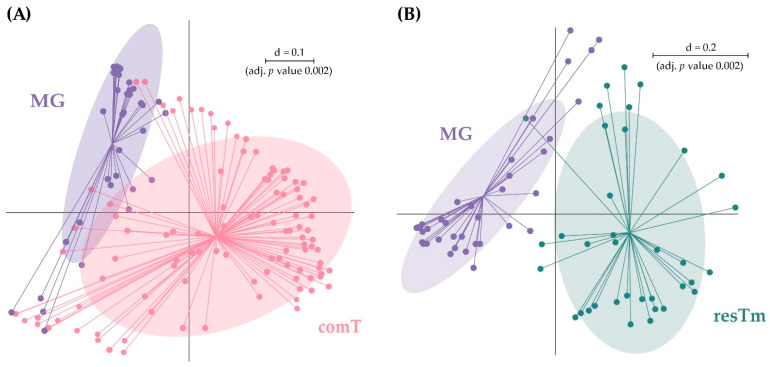
Pairwise β-diversity of the groups (**A**) MG and comT as well as (**B**) MG and resTm displayed as multidimensional-scaling (MDS) plot. The scale is an indicator for the differences in the phylogenetic makeup of microbiota between samples (β-diversity) based on general UniFrac distances (d = 0.1, 10% difference). comT, complete *Ixodes ricinus* tick; resTm, residual tick materials; MG, midgut.

When MG and comT samples were compared, the species richness for both effective richness and Shannon effective diversity in MG samples was significantly lower than in comT samples. The microbial profiles of MG samples clustered farthest from all other sample types; however, MG samples overlapped with comT samples (Figure 1B). As a result of a pairwise comparison, both MG and comT samples differed significantly in their microbial profile. Here, MG and comT samples seemed to overlap slightly (Figure 6A). MG samples were displayed on a different branch of the dendrogram than most comT; however, some outliers were observed (Figure 3). At taxonomic level, MG and comT samples differed significantly concerning various taxonomic levels as described in the Supplementary Table S4. Most important taxa displaying significant differences were the families Midichloriaceae (*p* ≤ 0.001) and Rickettsiaceae (*p* ≤ 0.001) with its genera *Candidatus* Midichloria (*p* ≤ 0.001) and *Rickettsia* (*p* ≤ 0.001), respectively, as well as the genus *Rickettsiella* (*p* ≤ 0.05). At the molecular strain level, MG and comT samples differed significantly regarding zOTU1 (*Candidatus* Midichloria mitochondrii, 100% similarity), zOTU2 (*Rickettsia helvetica*, 100% similarity), zOTU5 (*Williamsia maris*, 100% similarity), zOTU18 (Beijernickiaceae, 97.7% similarity), and zOTU31 (*Sphingomonas desiccabilis*, 97.7% similarity). In general, significant differences between comT and MG again need to be considered carefully, as MG samples represent pooled samples of three ticks and thus some bacterial species might appear overrepresented.

The α-diversity differed significantly in both effective richness and Shannon effective diversity among MG and resTm samples (Figure 1A). Interestingly, the microbial profiles of MG and resTm samples do not overlap except for one sample (Figure 6B). In general, β-diversity differed significantly between MG and resTm samples (Figure 6B). Again, this was most evident on the dendrogram, where most MG samples clustered on a branch without any resTm sample (Figure 3). Significant differences between MG and resTm samples at taxonomic level are displayed in the Supplementary Table S4. The most important taxa displaying significant differences were the family Midichloriaceae (*p* ≤ 0.001) with its genus *Candidatus* Midichloria (*p* ≤ 0.001). zOTU18 (Beijernickiaceae, 97.7% similarity), zOTU31 (*Sphingomonas desiccabilis*, 97.7% similarity), and zOTU5 (*Williamsia maris*, 100% similarity) were significantly stronger in resTm when compared to MG samples (Figure 5).

### 2.4. Salivary Gland Samples (SG)

**Figure 7:**
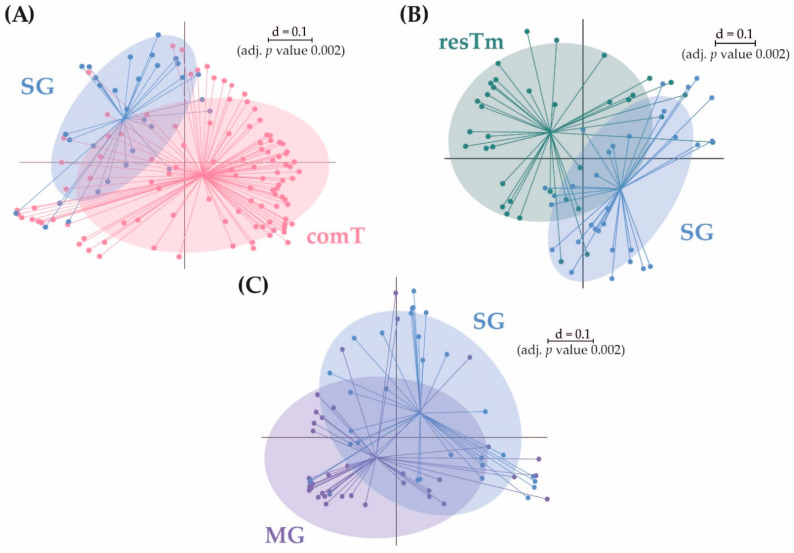
Pairwise β-diversity of the groups (**A**) comT and SG, (**B**) resTm and SG as well as (**C**) MG and SG displayed as multidimensional-scaling (MDS) plot. The scale is an indicator for the differences in the phylogenetic makeup of microbiota between samples (β-diversity) based on general UniFrac distances (d = 0.1, 10% difference). comT, complete *Ixodes ricinus* tick; resTm, residual tick material; MG, midgut; SG, salivary gland.

A mean of 7.5 effective species was calculated for SG samples. These samples displayed the second lowest α-diversity parameters (Table 1). As with the MG samples, the mean evenness of SG samples was 0.2, which again hints that a few species seemed to dominate this type of tissue. The microbial profiles in SG samples overlapped with all other sample types and clustered between MG samples and comT or resTm samples (Figure 1B). Pairwise β-diversity confirmed also that SG samples seemed to overlap with MG, resTm, as well as comT samples (Figure 7A–C). In the dendrogram, SG samples were randomly distributed on the upper two branches, while no SG sample clustered to the lowest branch (Figure 3). Again, Pseudomonadota (88.4%) was the most abundant phylum in this sample type. In contrast to all sample types described above, at family level, SG samples were dominated by Rickettsiaceae, followed by Midichloriaceae and Coxiellaceae in descending order. SG samples displayed the highest count of Anaplasmataceae (3.5%), while all other sample types showed abundances below 1.2% (Figure 2). Families listed above were represented by only one genus, respectively (i.e., *Rickettsia*, *Candidatus* Midichloria, and *Rickettsiella*). Further, for the genus *Pseudomonas,* an abundance above 5.0% was calculated. At molecular strain level, the genera *Rickettsia* and *Candidatus* Midichloria, again, were represented as described above by zOTU2 and zOTU1, respectively. However, we found another molecular strain for *Rickettsia* spp. with an abundance above 1.0% in SG samples (zOTU33, *Rickettsia felis*, 99.8% similarity). The genus *Rickettsiella* again was represented by the molecular strains zOTU3 (*Rickettsiella popilliae*, 98.4% similarity), zOTU6 (*Rickettsiella popilliae*, 98.7% similarity), and with a lower abundance zOTU34 (*Rickettsiella viridis,* 96.6% similarity). Similar to the other sample types, the genus *Pseudomonas* was represented by three different molecular strains with an abundance above 1.0% (zOTU16, *P. lurida*, 100% similarity; zOTU10, *P. poea*, 100% similarity; zOTU19, *P. laurylsulfativorans*, 100% similarity). Moreover, zOTU13 (*Candidatus* Neoerhlichia mikurensis, 100% similarity) represented the complete Anaplasmataceae family and zOTU20 (*Spiroplasma ixodetis*, 100% similarity) was observed with an abundance above 1.0% in SG samples.

Both effective richness and Shannon effective diversity were significantly lower in SG samples than in comT samples (Figure 1A). SG samples’ microbial profiles differed significantly from comT samples (Figure 7A), which is most obvious in the dendrogram, where no SG sample was aligned to the lower right branch containing most comT samples (Figure 3). Taxa differing significantly between SG and comT samples are shown in Supplementary Table S4, of which the genera *Rickettsia* and *Rickettsiella* (*p* ≤ 0.001 and *p* ≤ 0.05) seemed most important. According to the literature available, most other taxa displaying significant differences seemed to be associated to the environment. Comparing SG with comT samples at molecular strain level, significant differences were observed in zOTU18 (Beijernickiaceae, 97.7% similarity), zOTU2 (*Rickettsia helvetica*, 100% similarity), zOTU3 (*Rickettsiella popilliae*, 98.4% similarity), zOTU31 (*Sphingomonas desiccabilis*, 97.7% similarity), and zOTU5 (*Williamsia maris*, 100% similarity). In general, differences between comT and SG require careful interpretation, as SG samples represent pooled samples of three ticks and thus some bacterial species might appear overrepresented.

The α-diversity (i.e., effective richness and Shannon effective diversity) differed significantly between SG and resTm samples, and SG and resTm samples diverged significantly in their microbial profiles (Figure 7B). Significant differences at taxonomic level between SG and resTm samples are depicted in the Supplementary Table S4. Between these two groups, zOTU18 and zOTU5 differed significantly at molecular strain level (Figure 5).

The effective richness differed significantly, while Shannon effective diversity showed none among SG and MG samples. These groups displayed diverging microbial profiles (Figure 7C); again, this is most obvious in the dendrogram (Figure 3). In Supplementary Table S4, the difference in the relative abundances of the family Midichloriaceae (*p* ≤ 0.001) with its genus *Candidatus* Midichloria (*p* ≤ 0.001) seemed most important. The molecular strains zOTU1, zOTU18, zOTU31, and zOTU5 differed significantly between SG and MG samples (Figure 5).

In conclusion, the effective richness was highest in comT and resTm samples, as was the mean evenness, while SG and MG samples displayed a lower effective richness and evenness. MG samples displayed the lowest counts in effective richness (Table 1). In β-diversity, there were significant differences between all groups’ ecological profiles. All samples were dominated by the phylum Pseudomonadota. The most abundant family in all groups, except for SG samples, was Midichloriaceae. In SG samples, Rickettsiaceae were more abundant than Midichloriaceae. In comT, resTm, and MG samples, the second most abundant family was Pseudomonadaceae. Rickettsiaceae and Coxiellaceae were frequently present in resTm, MG, and SG samples. All samples contained zOTU1 except two, one comT sample which did not contain any zOTU1 at all and one SG sample which contained zOTU1 at a very low relative abundance.

### 2.5. Occurrence of Endosymbionts and Probable Pathogenic Species

As stated in the literature available to us, 16S rRNA gene amplicon sequencing is not able to soundly decipher the exact species level [41]. Therefore, species classification was assessed by comparing the molecular strains to well-characterized reference strains, and similarity was calculated based on the number of variations observed between the two strains [40]. Species described below will be addressed as “probable” endosymbionts or pathogens. The mock community included in this study contained several pathogens (i.e., *Borrelia burgdorferi* sensu stricto, *Borrelia afzelii*, *Borrelia garinii* subsp. *garinii*, *Borrelia garinii* subsp. *bavariensis*, and *Anaplasma phagocytophilum*), and molecular species that were identified with the help of the mock community indicate said species. Consequently, in tick samples, we assigned reads to various species either by their frequency of appearance and strain similarity (i.e., probable symbionts or commensals, *Candidatus* Midichloria mitochondrii, *Rickettsiella* spp., and *Spiroplasma ixodetis*), or by their appearance in both sample and mock community (i.e., for *Borrelia* spp. and *Anaplasma phagocytophilum*), or in the case of *Candidatus* Neoehrlichia mikurensis and *Rickettsia* spp. by strain similarity.

Considering probable symbionts and commensals, *Candidatus* Midichloria mitochondrii was observed in 99.5%, while the genus *Rickettsiella* was observed in 60.9% of all tick samples. *Spiroplasma ixodetis* was found in in 23.3% of all tick samples (Figure 3). *Candidatus* Midichloria mitochondrii was represented by one zOTU, *Rickettsiella* spp. and *Spiroplasma ixodetis* were represented by several zOTUs (Table 2).

The probable pathogenic species *Rickettsia* was observed in 41.1% of all tick samples. *Candidatus* Neoehrlichia mikurensis was found in 16.2%, *Borrelia* spp. in 12.4%, and *Anaplasma phagocytophilum* in 8.1% of all samples (Figure 3). The genus *Rickettsia* and *Borrelia* consisted of five different zOTUs, while *Anaplasma phagocytophilum* was assigned to four zOTUs. In contrast, *Candidatus* Neoehrlichia mikurensis consisted of only one zOTU (Table 3).

Describing the prevalence of probable endosymbionts and pathogens, only comT samples were considered, as all other tissue samples were pooled in three and thereby cannot represent the tick population accordingly. This resulted in a prevalence of 99.0% for *Candidatus* Midichloria mitochondrii, a prevalence of 72.6% for *Rickettsiella* spp., and a prevalence of 12.3% for *Spiroplasma ixodetis* in comT samples. The same applies for probable pathogenic species. A prevalence of 21.7% was observed for *Rickettsia* spp., a prevalence of 11.3% for *Candidatus* Neoehrlichia mikurensis, a prevalence of 5.7% for *Borrelia* spp., and a prevalence of 3.8% for *Anaplasma phagocytophilum* in comT samples.

## 3. Discussion

The aim of this study was to describe the BC of female *I. ricinus* ticks. In addition, bacterial communities in specific tick tissues were characterized. For this reason, midguts (MG), salivary glands (SG), and residual tick material without midgut and salivary glands (resTm) were obtained by microscope-aided dissection and analyzed in pools of three individuals. Overall, the quality of sequencing reads was highly satisfying, as read counts and rarefaction were highly comparable between the samples. However, we decided to apply the cutoff at a minimum of 10,000 reads to guarantee a reliable and comparable read quality in the final analysis and thus avoid biases. As a result, seven samples with fewer than 10,000 reads were excluded. comT samples contained all tissues such as MG, SG, and resTm, which were analyzed in separate tests. Thus, all bacteria found in the tissue samples are expected in comT samples. As the dissection of ticks is delicate and tissues could not always be separated from each other, we expected residuals of MG and SG in resTm samples. Whereas MG samples could be isolated with high certainty, SG samples might contain other tissues—for example, the Malpighian tubules. This assumption was confirmed by the data (Figure 7). As expected, bacteria associated with the environment occurred mostly in comT as well as in resTm samples, as these tissues had direct contact with the natural environment. This already has been described by Ross et al. (2018) [34] as have methods eliminating environment-associated bacteria (e.g., washing ticks in sterile water [34], 5.0% sodium hypochlorite, DNA away, Reactive Skin Decontamination Lotion (RSDL), 70% ethanol [42], or using Benzonase^®^ endonuclease [43]). In this study, these methods were omitted as our focus rested on the complete BC of ticks as encountered in the field. Moreover, environment-associated bacteria might allow conclusions on the tick’s location, especially concerning the geographical distribution of tick-borne pathogens [44]. In contrast, the environmental BC was problematic when analyzing low biomass samples (i.e., MG or SG samples) as the proportion of total bacterial DNA in these samples was already low and high amounts of environmental BC might be overamplified in the PCR steps. This could lead to a lower representation or even the non-detection of pathogens or symbionts that appear in low numbers during bioinformatic analysis. As a result, we decided to pool tick tissues (especially MG and SG) as already suggested by Ross et al. (2018) and Gall et al. (2016) [34,45]. We found several molecular bacterial strains with an abundance of more than 1.0% that, according to the literature available to us, might be associated with the environment: zOTU5 (*Williamsia maris*, 100% similarity), which was characterized as an ocean-associated bacteria [37]; zOTU15 (*Luteibacter anthropi*, 98.7% similarity), which was first isolated from human blood [46], while most other *Luteibacter* spp. are associated with the environment [47,48]; zOTU17 (*Variovorax paradoxus*, 100% similarity), which was described to be associated with soil and water [49]; zOTU18 (Beijerinckiaceae, 97.7% similarity), a microorganism that so far was identified only at the family level (accession number PAC0001013); zOTU26 (*Methylobacterium haplocladii*, 100% similarity), which was described to be associated with plants [50]; zOTU31 (*Sphingomonas desiccabilis*, 98.0% similarity) that was published to be associated with soil crust [38]; and zOTU36 (*Stenotrophomonas lactitubi*, 99.8% similarity), a bacterium that was isolated from surfaces with food contact [51], while other *Stenotrophomonas* spp. are associated with plants [39]. Further, zOTU42 (*Chryseobacterium lactis*, 99.6% similarity) was described to be associated with milk [52], whereas other *Chryseobacterium* spp. were linked to rhizosphere soil [53,54]. Lastly, zOTU53 (*Microbacterium liquefaciens*, 99.0% similarity) was isolated from environmental samples [55] and soil [56]. Fittingly, these molecular strains were mainly found in comT and resTm samples as already mentioned above. In MG and SG samples, however, environment-associated bacteria were observed at low abundances.

Another aim of this study was the identification of endosymbionts and pathogens within the ticks’ BC. Hence, a tick-specific mock community was designed. On one side, a mock community is used to identify possible deficiencies during DNA extraction, library preparation, and sequencing [57,58]. On the other hand, we anticipated to ease the identification of low-abundant tick pathogens such as *Borrelia* spp. and *Anaplasma* spp. The mock community was self-assembled with known strains; thus, we can surely comprehend the identity of bacterial strains in the mock community. The bioinformatic pipeline identifies the strains by comparing the sequences with a database, and thus strain identity on species level is only estimated. Both approaches in combination allow a precise identification of bacterial strains in the mock community and even allow a comprehension with the bacterial strains in project samples. In fact, this mock community allowed a precise identification of different pathogenic genera even at low abundances. Furthermore, we observed that the exclusion of the mock community had a negative effect on the occurrence of known pathogens. During analysis on IMNGS, an abundance cutoff of 0.25% was set, thus molecular strains with an abundance below this level were sorted out. As the mock community contained these molecular strains in high abundances, the cutoff was not applied for these species and known organisms were evident in the OTU table. Consequently, mock communities can serve as a helpful tool for the detection and identification of low-abundant bacteria in microbiome research [57]. Additionally, it is advisable to use an even more spectrum-enlarged mock community containing an even higher number of project-specific bacteria expected to be present at a low abundance. For tick samples, *Borrelia* spp. and *Anaplasma phagocytophilum* as well as bacteria not associated with ticks were used as a mock community. It might be advisable to further include *Rickettsia* spp., *Coxiella burnetii*, *Ehrlichia* spp., *Francisella tularensis*, or bacteria of the tick-borne relapsing fever complex in the mock community as these microorganisms may inhabit the vector tick. Here, we also added DNA from *Leptospira interrogans* to the mock community to serve as a negative control, since this spirochete has not been described to be taken up by ticks and consequently should not be detected in the BCs of ticks.

A primary focus of the project was the goal to identify and characterize possible endosymbionts within the ticks’ BC. In all ticks with the exception of one sample, we found *Candidatus* Midichloria mitochondrii (zOTU1) in high abundances, which suggests that this bacterium might play an essential—possibly endosymbiontic—role in the BC of *I. ricinus* ticks. This has already been described multiple times [59,60]. *Candidatus* Midichloria mitochondrii first has been reported to be present in *I. ricinus* ticks in 2004 [60] and is thought to reside in the mitochondria of tick cells. Furthermore, this endosymbiont is presumed to have a mutualistic relationship with the tick. *Candidatus* Midichloria mitochondrii holds genes for biosynthesis of B vitamins as well as *cbb_3_*-type cytochrome *c* oxidase and might be a source for ATP under low-oxygen conditions [61,62]. In female ticks, an abundance of almost 100% was described in ovary cells but without major impact on reproduction, whereas in male ticks an abundance of only 40% was reported [63].

*Rickettsiella* spp. have been described as facultative, intracellular symbionts within ticks as appropriate host [36]. *Rickettsiella popilliae* (zOTU3, 98.4% similarity and zOTU6, 98.7% similarity) and *Rickettsiella grylli* (zOTU59, 98.4% similarity) have already been described in ticks [64] and were also present in this data set. *Rickettsiella grylli* is thought to be probably pathogenic to mammals due to its low host specificity and can infect mammals even via inhalation [65]. Additionally, we found *Rickettsiella viridis* (zOTU34, 96.6% similarity) and *Rickettsiella isopodorum* (zOTU43, 100% similarity). *Rickettsiella* spp. are represented in all life stages of *I. ricinus* ticks as described by Garcia-Vozmediano et al. (2021) [36]. Survival of ticks in subsequent life stages could probably be improved with the presence of *Rickettsiella* spp. Depending on the geographical location, there might be a broad genetic variability of *Rickettisella* spp. Another biological characteristic of *Rickettsiella* spp. is the manipulation of its attractiveness to predators and parasitoids [66]. Coinfections between *Candidatus* Midichloria mitochondrii and *Rickettsiella* spp. were published as the most frequent coinfections in ticks throughout Europe [36].

*Spiroplasma ixodetis* (zOTU20, 100% similarity and zOTU87, 98.8% similarity) was firstly described in *I. pacificus* ticks in the US in 1995 as an intracellular microorganism [67]. Similar *Spiroplasma* spp. isolated from *I. ricinus* in Germany, have in the meantime been described by Henning et al. (2006) [68]. *Spiroplasma* spp. have been isolated from unfed adult *I. ricinus* ticks of both genders and from tick eggs, which indicates that these microorganisms might be endosymbionts [69]. However, there are some strains pathogenic for plants, insects, and even humans [70,71]. *Spiroplasma mirum* (zOTU460, 99.6% similarity) has been reported to induce cataract in mice [72] and seldomly in humans [73], whereas for *Spiroplasma* spp. infection in general was associated with transmissible spongiform encephalopathy in ruminants and humans [74]. Nevertheless, spiroplasmal infectivity and pathogenicity still need more investigation. Apparently, there is a negative association between the occurrence of *Borrelia* spp. and *Spiroplasma* spp. in ticks, as well as between *Spiroplasma* spp., *Rickettsia* spp., and *Candidatus* Neoehrlichia mikurensis [29]. Named pathogens were found in our study as well.

*Bb*sl is widely known as an agent of LB [13] in humans and animals with a seroprevalence of 9.4% in humans [75] and 22.2% in dogs [76]. Here, we reported a *Bb*sl prevalence of 5.7% in *I. ricinus* ticks, which is comparable to other studies with prevalence varying from 6.1% to 20.0% in Germany [36,77,78].

Another emerging pathogen, *Rickettsia helvetica*, belongs to the spotted fever group rickettsiae. The primarily called “Swiss Agent” was described by Burgdorfer et al. (1979) [79] for the first time and was confirmed as *Rickettsia helvetica* in 1993 [80]. In the literature, *Rickettsia helvetica* is presumed to be pathogenic for humans [81,82,83]. Contrary, *Rickettsia* spp. are assumed to be symbionts to the ticks as they might provide nutrients such as folate. This endosymbiotic support is necessary as the ticks’ blood meal does not supply B vitamins [84]. In this work, a prevalence of 19.8% was calculated for *Rickettsia helvetica* in *I. ricinus* ticks. In dogs, a seroprevalence of 66.0% was reported in Germany [85], while in ticks, prevalence varied between 12.0% and 99.8% [86,87,88].

Further, the intracellular microorganism *Candidatus* Neoehrlichia mikurensis was published by Kawahara et al. in 2004 [89] for the first time, but probably was described by Schouls et al. in 1999 [90] and by Pan et al. in 2003 [91] in the past. This still uncultured bacterium causing neoehrlichiosis has been found in *I. ricinus* ticks already [92]. In humans, the first case reports regarding infections with *Candidatus* Neoehrlichia mikurensis were published in 2010 [93,94,95]. To the best of our knowledge in the literature, only one case report exists where this bacterium was isolated from a dog [96]. In this examination, a prevalence of 11.3% was calculated for *I. ricinus* ticks, while other authors described a prevalence of 24.0% in Germany [92] or 4.2% in Austria [97].

Tick-borne fever in sheep was described in 1932 [98], and in 1949 the infectious agent *Rickettsia phagocytophilum* was identified firstly [99]. Later, this microorganism was reordered and renamed to *Anaplasma phagocytophilum* [100]. Currently, *Anaplasma phagocytophilum* is further reported as the agent of human, canine, and equine granulocytic anaplasmosis and is present in intracytoplasmatic vacuoles [100]. In this study, the prevalence of *Anaplasma phagocytophilum* in *I. ricinus* ticks was 3.8%. The literature showed a similar prevalence between 3.6% and 11.6% [101,102]. In dogs, a seroprevalence of 43.0% was reported for Germany [103], while in humans, a seroprevalence of 8.7% was calculated [104].

In conclusion, this work describes a new approach of dissecting ticks and isolating tick tissue, extracting tick DNA using an automated DNA extraction method, and the use of a tick-specific mock community to identify tick-specific pathogens and endosymbionts. With this method, we were able to identify ticks’ endosymbionts described by other authors, as well as pathogens known to be TBD. An advantage of this new, automated DNA extraction protocol is its easy and time-friendly handling. Further, as all steps of DNA purification are conducted mechanical, the contamination and human error are reduced in these steps. This is very important when handling low biomass samples. This approach and further recommendations for using a tick-specific mock community—possibly even a computational constructed mock community—allow the observation of tick populations regarding the prevalence of pathogens in a certain area as well as the development of pathogen abundances. This approach might further be used as a possibility to assess the danger of TBD infections in certain forest and park areas and thus might allow a hazard assessment and specific premonition.

## 4. Materials and Methods

### 4.1. Tick Sampling

A total of 210 ticks were collected in autumn 2021 (September and October; Table 4) using the flagging method. For this purpose, a white flannel sheet was dragged slowly over pasture, woodland, and vegetation. Only female *I. ricinus* ticks were collected from three different locations in Germany: Grendacher forest, a private woodland near Traunstein, Kranzberger, a state forest near Freising, and Schleißheimer Schlosspark in Oberschleißheim, a public park near Munich. All areas are risk areas for Lyme borreliosis and tick-borne encephalitis virus [105]. Ticks were collected from the sheet with forceps and were placed into tubes separately. The dissection of the ticks was conducted directly the day after collection avoiding possible microbiome shifts (e.g., due to starvation of the tick or prolonged periods of cold temperatures). Until then, the samples were stored at 4 °C.

### 4.2. Dissection of Female Ixodes ricinus Ticks

Dissection of 104 female ticks was conducted using a stereomicroscope with a magnification of up to 120× and depth of focus (Leica M205 C with FusionOptics, Leica Microsystems GmbH, Wetzlar, Germany). First, ticks were fixed on top of a microscope slide using super glue (UHU Sekundenkleber Pipette, UHU GmbH & Co. KG, Bühl, Germany; Supplementary Figure S1). While the glue dried, tubes for the three different tissues (i.e., resTm, MG, and SG) were prepared with 100 µL sterile-filtered phosphate-buffered saline (PBS, Carl Roth, Karlsruhe, Germany). The scutum of the tick was removed using a scalpel blade (No. 11, Ribbel Import-Export GmbH & Co. KG, Wuppertal, Germany) and inserted into the tube for residual tick material (resTm; Supplementary Figure S2). As the tick’s internal tissues were accessible after removing the scutum, the MG and SG were removed next with jewelers’ forceps (Dumont No. 5, Merck KGaA, Darmstadt, Germany) (Supplementary Figure S3) and transferred into corresponding tubes prefilled with PBS. The residual tissues of the tick, such as legs, capitulum (head), remaining tissue, and idiosoma (body) were further added to the residual tick material tube (resTm). All dissected tissue samples were processed in pools of three to increase input biomass, sequencing quality, and to reduce noise from reagent-based or environmental contamination [34]. Samples were stored at −30 °C until DNA isolation.

### 4.3. DNA Extraction

Complete ticks (comT samples; *n* = 106) were crushed multiple times using a scalpel blade. Afterward, crushed ticks were transferred into a lysing matrix tube (Lysing Matrix tube D, 2.0-mL tube, MP Biomedicals, Eschwege, Germany). A total of 300 µL of Incubation Buffer (D920B-C, Promega GmbH, Walldorf, Germany) were added then. All samples were transferred into the FastPrep-24™ device (MP Biomedicals) and homogenized in one cycle of 5.5 m/s for 30 s. After the homogenization step, 30 µL of 20-mg/mL Proteinase K (included in AS1290, Promega GmbH) and 200 µL of Lysis Buffer (included in AS1290, Promega GmbH) were added to the sample. After mixing (Vortex-Genie 2, Scientific Industries Inc., New York, NY, USA) and ten seconds of centrifugation at 10,000× *g* (Eppendorf Centrifuge 5430, Eppendorf SE, Hamburg, Germany), the samples were incubated at 56 °C and 350 rpm for a minimum of two hours on the shaker (ThermoMixer C, Eppendorf SE). Next, 5 µL of 10-mg/mL RNase A (Thermo Fisher Scientific, Waltham, MA, USA) were added and the samples were incubated for another 20 min at 37 °C and 350 rpm on a shaker (ThermoMixer C, Eppendorf SE). Then, 300 µL of Lysis Buffer (Promega GmbH) were added to each sample. The mixing (Vortex-Genie 2, Scientific Industries Inc.) and centrifugation steps were repeated as described above. Dissected tick samples (*n* = 104) were transferred into a tube with 300 µL Incubation Buffer (D920B-C, Promega GmbH), then 30 µL of 20-mg/mL Proteinase K (included in AS1290, Promega GmbH) and 200 µL of Lysis Buffer (included in AS1290, Promega GmbH) were added to the sample. Afterward, samples were mixed (Vortex-Genie 2, Scientific Industries Inc.) and centrifugated for ten seconds until reaching 10,000× *g* (Eppendorf Centrifuge 5430, Eppendorf SE). Thereafter, the samples were incubated at 56 °C and 350 rpm for a minimum of two hours on the shaker (ThermoMixer C, Eppendorf SE). Next, 5 µL of 10-mg/mL RNase A (Thermo Fisher Scientific) were added, and the samples were incubated for another 20 min at 37 °C and 350 rpm on a shaker (ThermoMixer C, Eppendorf SE). Afterward, 300 µL of Lysis Buffer (Promega GmbH) were added to each sample. The mixing (Vortex-Genie 2, Scientific Industries Inc.) and centrifugation steps were repeated as described above. Furthermore, negative controls testing the dissection work (especially the instruments), the cartridge of the MaxWell 16 MDx purification system, and all reagents were prepared according to the tick tissue samples. Complete *I. ricinus* ticks, dissected tick samples, and negative controls were transferred into the MaxWell 16 LEV Blood DNA Kit cartridges (included in AS1290, Promega GmbH), and the automatized DNA purification process using the Maxwell 16 MDx (Promega GmbH) was conducted. The samples were eluted in 60 µL Elution Buffer (included in AS1290, Promega GmbH). After DNA extraction, DNA content was measured using BioPhotometer (D30, Eppendorf SE). Samples were stored at −30 °C until further processing. An amount of 20 µL DNA was shipped to Eurofins Genomics laboratory (Eurofins Genomics GmbH, Ebersberg, Germany) where sequencing of the 16S rRNA gene was performed.

### 4.4. Design and Creation of the Mock Community

Seven bacterial species and strains available at the Chair of Bacteriology and Mycology of LMU Munich, Germany were individually thawed, cultured, and DNA was isolated to construct a mock community. The DNA for *Anaplasma phagocytophilum* and *Leptospira interrogans* was isolated from previous experiments and thus only thawed and reused. For DNA extraction, the MaxWell 16 MDx purification system was used following the same DNA extraction protocol as described for dissected tick samples. Afterward, the isolated DNA was pooled to construct the mock community. Four different *Borrelia* genospecies (Table 5) were used to possibly identify *Borrelia* spp. occurrence in the prepared tick samples. *Anaplasma phagocytophilum* was added as it represents another pathogenic bacterium other than *Borrelia* spp. appearing in *I. ricinus* ticks. As a common intestinal bacterium in the 16S rRNA microbiome analysis *Escherichia coli* was added to the mock community because it is easy to detect. As negative control, the spectrum of the self-designed mock community was enlarged with *Leptospira interrogans* as this bacterium should not be present in a tick sample.

### 4.5. Bacterial 16S rRNA Gene Sequencing

The BC of extracted tick samples and tissue samples was sequenced at Eurofins Genomics laboratory (Eurofins Genomics GmbH) by targeting the hypervariable V1–V3 region of the 16S rRNA gene (primers: 27F: 5′-AGA GTT TGA TYM TGG CTC AG-3′ and 519R: 5′-GTA TTA CCG CGG CKG CTG-3′ [106]). A two step-PCR was executed preparing the samples for sequencing [107]. The first PCR was conducted to amplify the 16S rRNA target gene (V1–V3 region). The second PCR was conducted to barcode the amplified PCR products of the first PCR generating a DNA library. Here, Illumina adaptor sequences were added to the PCR primers. The DNA concentrations were measured by a fluorometric method. The final pool was sequenced in paired-end mode (2 × 300 bp) and pipetted into the well of the Illumina cartridge using MiSeq Reagent Kit v3.

### 4.6. Data Analysis

16S rRNA gene amplicon data were analyzed as described previously [108] with the following changes. As sequencing data arrived demultiplexed, data were merged using a Perl script from the IMNGS website (https://www.imngs.org/static/files/remultiplexor.zip; accessed on 22 August 2022) generating I1, R1, and R2 files. These files were then checked for quality using FastQC [109], and sequence quality per base for R1 and R2 has been provided as Supplementary Figures S4 and S5. The named multiplexed fastq files were then processed using the “Integrated Microbial Next-Generation Sequencing” (IMNGS) pipeline [110], a UPARSE-based platform [111]. Sequences were demultiplexed, with a quality trimming score of less than 15, and paired. Paired reads with an expected error of less than two and sequences with a length smaller than 300 and higher than 600 nucleotides were excluded from the further analysis. To prevent analysis of the regions with distorted base composition observed at the start of sequences, the remaining reads were trimmed with a trimming length of ten nucleotides on each end. The presence of chimeras was tested with UCHIME [112]. IMNGS supplies both operational taxonomic units (OTUs) and denoised zero-radius operational-taxonomic units (zOTUs) for different parts of the analysis. OTUs were clustered at 97% sequence similarity [110], while zOTUs were calculated using UNOISE 2 [113] from the USEARCH 11 package [114]. Only those OTUs and zOTUs occurring with at least 0.25% relative abundance in at least one sample were kept for further analysis. Taxonomy was assigned at an 80% confidence level with SILVA [115]. Following the processing on the IMNGS platform, samples with a read count lower than 10,000 were excluded from subsequent analysis (Supplementary Figure S6). Thereafter, IMNGS steps were repeated with the parameters described above. Afterward, the data provided by IMNGS were refined by editing the phylogenetic tree and taxonomy using SILVA [115]. Sequences were aligned anew, and phylogenetic trees were constructed using the neighbor-joining method available on the software MEGA-X version 10.1.8 [116]. Taxonomy, which was available only at kingdom, phylum, class, or order level was confirmed using the 16S-based ID provided by EzBioCloud and if possible adapted [40]. All mentioned molecular strains (zOTUs) were compared to the EzBioCloud-database and strain similarity based on the variations on sequence level is indicated in brackets behind the corresponding zOTU. Moreover, the taxonomy was revised according to the nomenclature provided by the LPNS database [117] using the phylum names introduced in 2021 [118]. Since this leads to a renaming of all phyla occurring in this study, the former names are given in parentheses. All downstream analyses were carried out using Rhea [119], a modular pipeline for microbial profiling of 16S rRNA gene amplicon sequencing data in an R programming environment (R 3.6.3, R Foundation for Statistical Computing, Vienna, Austria) as described previously [119]. The pipeline is available on the GitHub repository (https://github.com/Lagkouvardos/Rhea; accessed on 25 August 2022). The OTU and zOTU tables of all experimental samples can be found in the Supplementary Material (Supplementary Tables S1 and S2). For the α-diversity, the effective richness (i.e., effective number of species) and the effective Shannon diversity were calculated using OTUs [120]. All further parameters were calculated using zOTUs. Based on generalized UniFrac distances, β-diversity was calculated [121]. *p* values were corrected for multiple testing using the Benjamini–Hochberg method [122]. For statistical testing, taxa with a prevalence equal to or more than 20% (proportion of samples positive for the respective taxa) in at least one of the groups and a relative abundance of equal to or more than 0.25% were considered. For multiple groups, a Kruskal–Wallis rank sum test was applied. Afterward, the Wilcoxon rank sum test was used for pairwise comparisons. A non-linear Fisher exact test was used to determine the differences between samples with a low prevalence. Data were visualized using Illustrator CS6 version 16.0.0 (Adobe Inc., San José, CA, USA). The most abundant taxa were visualized using the software Prism, version 2010 (GraphPad Software, San Diego, CA, USA). The dendrogram was designed using the free available webserver Evolview v3 [123]. Concerning the mock community, it seemed that the exclusion of the mock community had a detrimental effect on low abundant pathogenic bacteria. Without the mock community in the final analysis, the abundance cutoff value shifted in such way that especially *Borrelia* spp. were no longer displayed in the samples. Consequently, we again included the mock community in final analysis as we recommended above for studies using ticks.

## Figures and Tables

**Table 1:** Read count and α-diversity (effective richness) shown as average mean.

Sample	Read Count	Effective Richness
comT	34,392	32.8
resTm	38,212	28.3
MG	43,238	4.9
SG	35,674	7.5

comT, complete *I. ricinus* tick; resTm, residual tick material; MG, midgut; SG, salivary gland.

**Table 2:** Probable endosymbiotic species detected in *Ixodes ricinus* ticks and their assigned zOTUs.

Species	zOTUs
*Candidatus* Midichloria mitochondrii	zOTU1 (*Candidatus* Midichloria mitochondrii, 100% similarity)
*Rickettsiella* spp.	zOTU3 (*Rickettsiella popilliae*, 98.4% similarity)
	zOTU34 (*Rickettsiella viridis*, 96.6% similarity)
	zOTU43 (*Rickettsiella isopodorum*, 100% similarity)
	zOTU59 (*Rickettsiella grylli*, 98.4% similarity)
	zOTU6 (*Rickettsiella popilliae*, 98.7% similarity)
*Spiroplasma ixodetis*	zOTU20 (*Spiroplasma ixodetis*, 100% similarity)
	zOTU87 (*Spiroplasma ixodetis*, 98.8% similarity)

**Table 3:** Probable pathogenic species detected in *Ixodes ricinus* ticks and their assigned zOTUs.

Species	zOTUs
*Rickettsia* spp.	zOTU108 (*Rickettsia monacensis*, 100% similarity)
	zOTU173 (*Rickettsia bellii*, 99.6% similarity)
	zOTU2 (*Rickettsia helvetica*, 100% similarity)
	zOTU226 (*Rickettsia* spp., 94% similarity)
	zOTU33 (*Rickettsia felis*, 99.8% similarity)
*Candidatus* Neoehrlichia mikurensis	zOTU13 (*Candidatus* Neoehrlichia mikurensis, 98.4% similarity)
*Borrelia* spp.	zOTU968 (*Borrelia miyamotoi*, 98.6% similarity)
	zOTU1616 (*Borrelia garinii* subsp. *garinii*, 100% similarity)
	zOTU1977 (*Borrelia burgdorferi* sensu stricto, 100% similarity)
	zOTU3122 (*Borrelia afzelii*, 100% similarity)
	zOTU1296 (*Borrelia garinii* subsp. *bavariensis*, 100% similarity)
*Anaplasma phagocytophilum*	zOTU364 (*Anaplasma phagocytophilum*, 99.8% similarity)
	zOTU370 (*Anaplasma phagocytophilum*, 99.8% similarity)
	zOTU405 (*Anaplasma phagocytophilum*, 99.6% similarity)
	zOTU656 (*Anaplasma phagocytophilum*, 99.6% similarity)

**Table 4:** Overview of the prepared samples.

Sample Type	Number of Samples
Collected female *Ixodes ricinus* ticks	210
Dissected tick samples	104
Midgut samples (MG) *	37
Salivary gland samples (SG) *	31
Residual tick material samples (resTm) *	36
Complete tick samples (comT)	106

* in pools of three.

**Table 5:** Species and amount of gDNA used for mock community.

Species	Amount of gDNA Used (ng)
*Borrelia burgdorferi* sensu stricto N40 P2	12
*Borrelia afzelii* P Per P1	12
*Borrelia garinii* subsp. *garinii* P Be P1	12
*Borrelia garinii* subsp. *bavariensis* P Bn P1	12
*Anaplasma phagocytophilum*	12
*Escherichia coli*	12
*Leptospira interrogans* LHJ P93	12

## Data Availability

The data that support the findings of this study are available in the Sequence Read Archive under the reference number PRJNA908769.

## Supplementary Materials

The supporting information can be downloaded at: https://www.mdpi.com/article/10.3390/ijms24021100/s1.
