# Supplementary material for: Revealing the Tick Microbiome: Insights into Midgut and Salivary Gland Microbiota of Female Ixodes ricinus Ticks
Source: Int J Mol Sci. 2023 Jan 6;24(2):1100. doi: 10.3390/ijms24021100 (PMC9864629; doi:10.3390/ijms24021100)
Supplement: Supplementary file 1 [file ijms-24-01100-s001.zip › Supplementary Figures S1-S6.pdf]

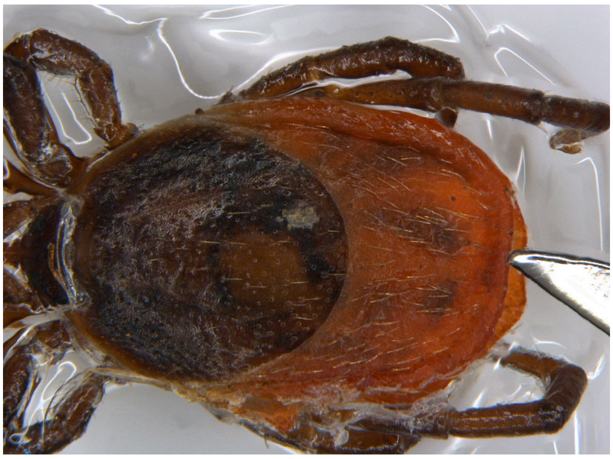

Figure S1: *I. ricinus* tick (fixed on a microscope slide; 30× magnification).

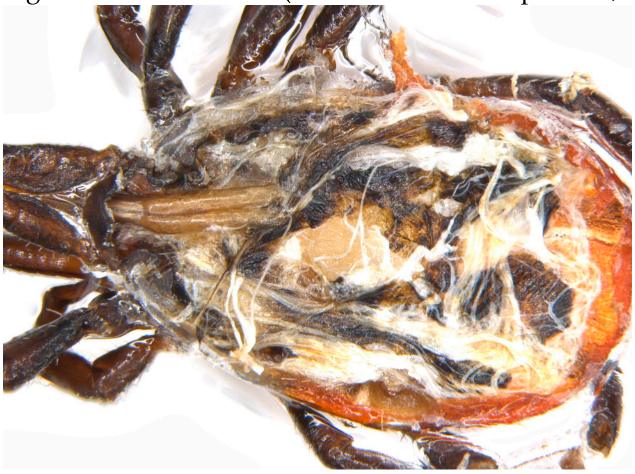

Figure S2: *I. ricinus* tick after removing the scutum (fixed on a microscope slide; 30× magnification).

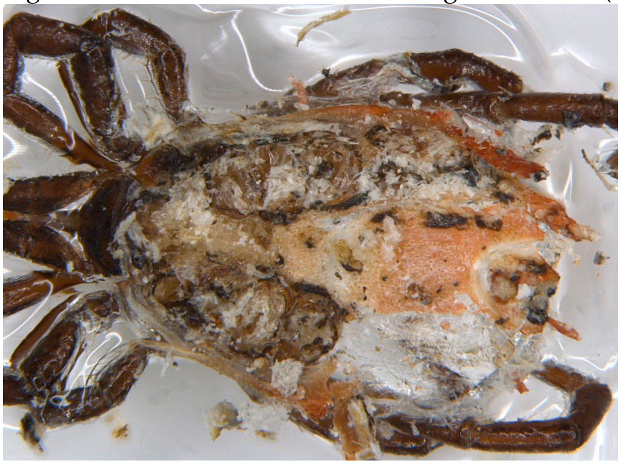

Figure S3: *I. ricinus* tick after removing midgut and salivary glands (fixed on a microscope slide; 30× magnification).

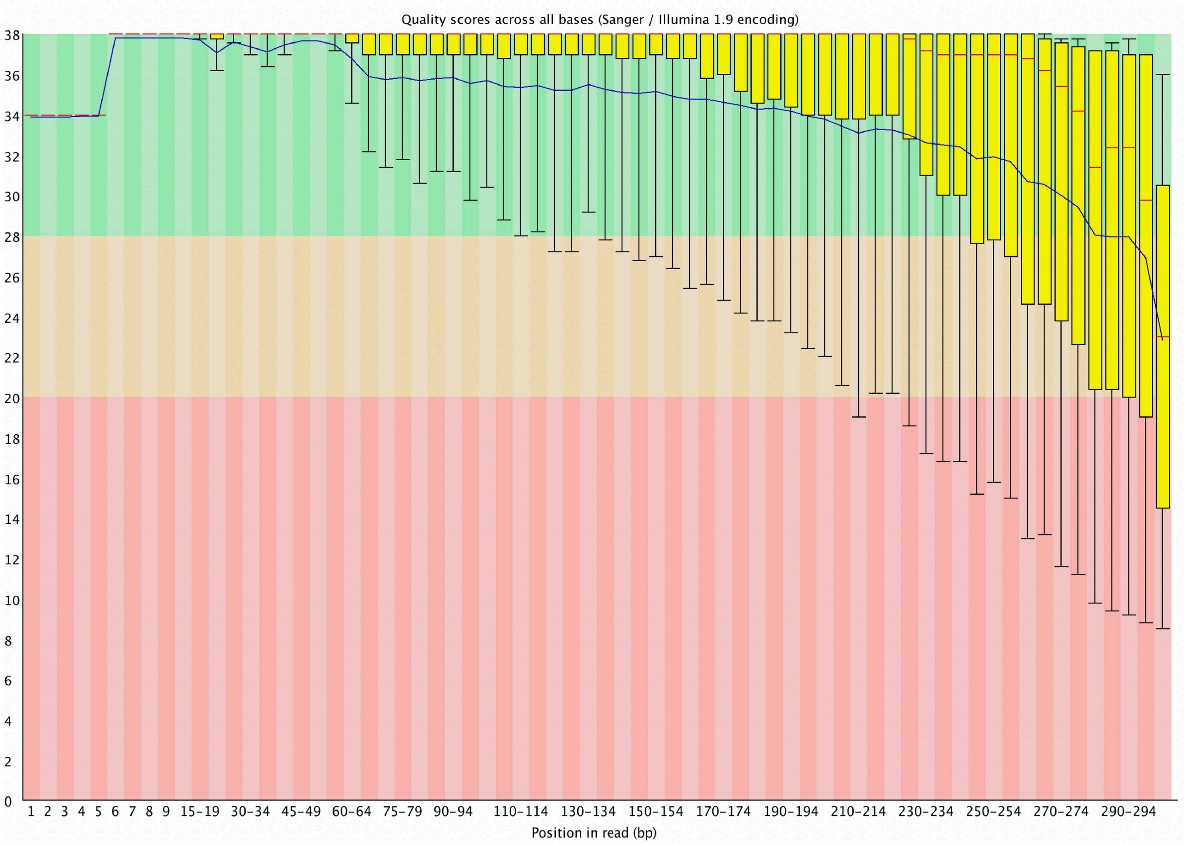

Figure S4: Sequence quality per base for R1.

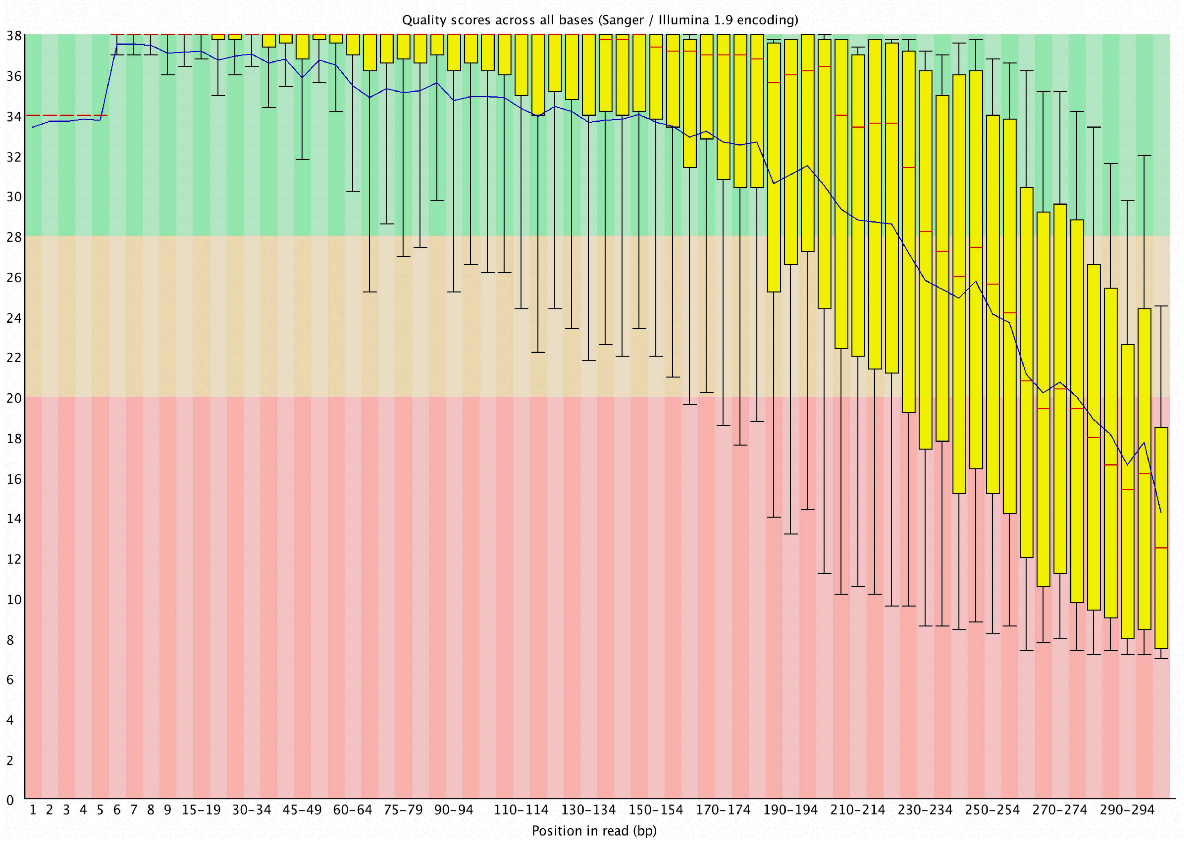

Figure S5: Sequence quality per base for R2.

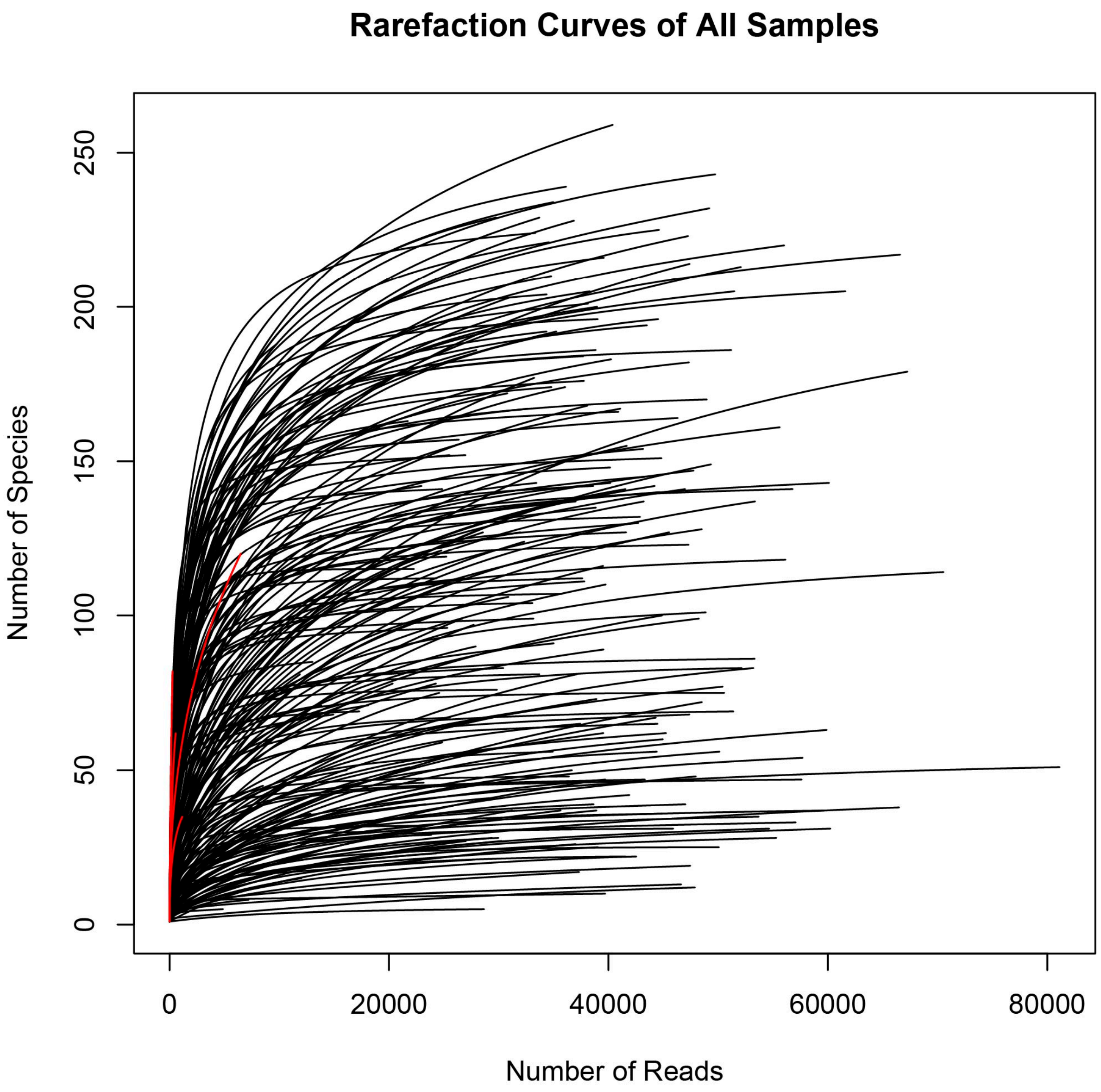

Figure S6: Rarefaction curve before removal of low-quality sequences.
